# Design optimization of large-scale bifacial photovoltaic module frame using deep learning surrogate model

**DOI:** 10.1038/s41598-024-64594-4

**Published:** 2024-06-25

**Authors:** Dongwoon Han, Seongtak Kim

**Affiliations:** Gangwon Technology Application Division, Korea Institute of Industrial Technology, Wonju, 26336 Republic of Korea

**Keywords:** Engineering, Energy harvesting

## Abstract

Recently, the wafers used in solar cells have been increasing in size, leading to larger module sizes and weights. The increased weight can cause deflection of photovoltaic (PV) module, which may lead to decreased cell efficiency. In this study, we developed a deep neural network (DNN)-based finite element (FE) surrogate model to obtain the optimal frame design factors that can improve deflection in large-scale bifacial PV module. Initially, an FE model was constructed for large-scale bifacial PV module. Based on this, the FE surrogate model was trained using 243 FEA datasets generated within the proposed range of factors. Furthermore, it was improved through Bayesian optimization and k-fold validation. As a result, the final loss value was $3.743 \times 10^{-4}$, and the average mean absolute percentage error (MAPE) and coefficient of determination ($R^2$) values for deflection and weight were 0.0017, 0.9972 for the training set, and 0.0020, 0.9962 for the test set, respectively. This indicates that the trained FE surrogate model possesses significant accuracy. After generating 1 million datasets within the range of frame design factors, the trained model was used to obtain predictions. Based on this data, the frame design factors that minimize both deflection and weight were identified as about a = 1.5, b = 13.7, c = 1.5, d = 3.0, e = 4.3. At this point, the deflection was 11.1 mm, and the weight was 3.6 kg. After altering the frame shape with the derived factors, FEA was conducted. The results matched for both deflection and weight, with almost no error. At this point, the weight increased by approximately 12.8% compared to the existing, while the deflection decreased by about 9.6%. Additionally, we analyzed the relationship between deflection and weight for each factor and secured the basis for the derived results. Consequently, our FE surrogate model accurately predicted the FEA results and quickly identified the optimal factors that minimize deflection and weight.

## Introduction

Recently, development to increase the area of wafers used in solar cell manufacturing is accelerating around the world. When wafer surface area is increased, it leads to higher unit production and output. Additionally, during module manufacturing, it becomes possible to compose the module with a relatively smaller number of cells on the same area, resulting in advantages such as reduced electrical components and assembly time per cell, ultimately lowering production costs. Before 2019, M2 cells accounted for over 80% of the wafer market share. However, by 2021, M6 cells constituted 42%, M10 cells 27%, and as of the second quarter of 2022, it is predicted that M10 cells will surpass 50%. This trend indicates a rapid increase in the market share of large silicon wafers^1^. Furthermore, with the wafer surface area increasing, there is ongoing development of module integration incorporating large-scale solar cells to meet the demand for high-output module. This has led to a gradual increase in the size of module^2^. However, as the size of PV module increases, so does their weight (*w*). Consequently, when applying conventional commercial aluminum (Al) frames for installation, there is a possibility of module deflection ($\delta$) due to wind pressure, snow load, and self-weight. If the module undergoes deformation due to the load, it may lead to cracking or delamination of ribbons inside the module, resulting in an increase in dead cells. This becomes a contributing factor to a decrease in module fill factor and conversion efficiency^3,4^. Research on the deformation behavior and shape optimization of module under mechanical loads is being conducted globally. Hartley et al.^5^ conducted a study on both monofacial and bifacial PV module. They developed a three-dimensional FE model through a comparison and validation of gravity experiments and FEA. The study identified key parameters influencing module deformation. Noh et al.^6^ analyzed module deformation and cell damage tendencies for monofacial large-scale PV module through FEA and mechanical load testing. Tummalieh et al.^2^ proposed a suitable frame shape for monofacial large-scale PV module considering mechanical, photoelectricity, cost, and lifespan aspects. Meanwhile, alongside the wafer surface passivation, rapid developments are taking place in the field of bifacial cells and module. Currently, for bifacial cells, the rear efficiency can reach up to a maximum of 90%, compared to the front efficiency, which is at 60%^7^. Moreover, for bifacial module, although the production cost is slightly higher compared to monofacial module, they offer the advantage of increased energy production, ranging from 1.25 to 1.3 times more^8–10^. In 2020, bifacial cells accounted for a market share of 20%, and it is predicted to rise to 70% by 2030. Bifacial modules had a market share of 12% in 2020, and it is anticipated to increase to 30% by the year 2030^11^. Bifacial modules, with a glass backsheet, are relatively heavier compared to monofacial modules. Therefore, when subject to increased surface area, there is a higher likelihood of frame deformation due to self-weight, leading to potential module deflection. However, research on design optimization for large-scale bifacial PV module frames is currently lacking. Therefore, in this study, we aim to analyze the deflection of large-scale bifacial PV module using FEA. Additionally, we aim to develop a DNN-based FE surrogate model to derive the PV module frame design factors that minimize both deflection and weight. Furthermore, by analyzing the impact of each factor on deflection and weight, we intend to provide guidance for future PV module frame design.

## Results and discussion

**Figure 1:**
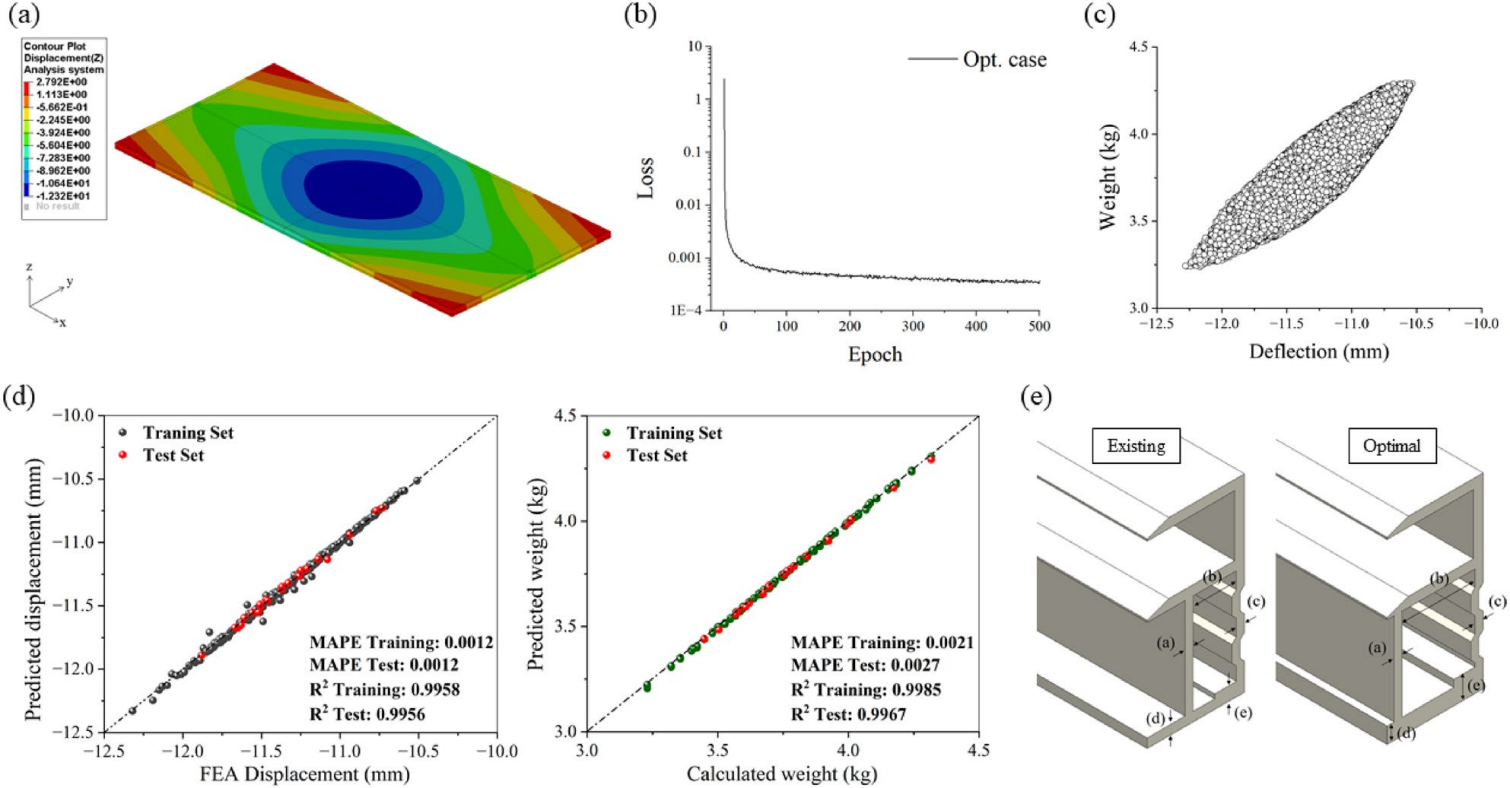
(**a**) Displacement distribution of large-scale PV module. (**b**) Loss–Epoch graph at the optimal hyperparameter combination (**c**) The relationship between deflection and weight through DNN predictions for 1 million datasets. (**d**) Linear comparison of training set and test set: FEA results vs. FE surrogate model predictions. (**e**) Frame shape before and after optimization.

Figure 1a shows the FEA results of the large-scale bifacial PV module with a commercially available Al frame. The analysis revealed that the maximum deflection occurred at the center of the module, confirming that this behavior is consistent with previous studies on the mechanical loading of PV module^2,5,6,12,13^. At this point, the maximum deflection of PV module was 12.3 mm, and the weight of frame was 3.2 kg, with a displacement of up to approximately 2.8 mm in the opposite direction occurring due to the reaction force caused by deflection from the support point to the end of the module. FEA took approximately 4800 s to calculate the deflection for the given case using a Xeon Gold 6434 CPU (3.7 GHz). Among a total of 243 cases, the minimum deflection was observed in condition 243 at 10.5 mm, indicating a reduction of about 14.6% in deflection compared to when using the conventional Al frame. At this time, the frame weight increased to 4.3 kg, which is an approximate 34.4% increase. Excessive deflection in the module can lead to cell damage, resulting in decreased efficiency^14^. While making the frame thicker can relatively reduce deflection, it also increases the production cost. Therefore, it is important to appropriately adjust the frame thickness to secure stiffness and reduce the deflection occurring in the module. Based on the FE surrogate model trained via the DNN algorithm, we derived frame design factors that minimize both weight and the amount of deflection.

The hyperparameter values that minimize the loss function, derived through Bayesian optimization, are as follows: the number of hidden layers is 4, the number of nodes in the hidden layer is 118, the batch size is 1, and the learning rate is 4.557 x $10^{-2}$. Based on the derived hyperparameters, the model was retrained. The graph of loss per epoch, as shown in Fig. 1b, indicates that the final loss value was 3.743 x $10^{-4}$. The Fig. 1d represents a linear comparison between the FEA results and the predictions of the FE surrogate model trained based on Bayesian optimization, for both deflection and weight. The slope of the linear comparison graph for both deflection and weight approximated 1, indicating that the trained FE surrogate model accurately predicts the analysis results. Additionally, the MAPE values and the $R^2$ values were 0.0012, 0.9958 for deflection and 0.0021, 0.9985 for weight in the training set, respectively. In the test set, the MAPE and $R^2$ values were 0.0012, 0.9956 for deflection and 0.0027, 0.9967 for weight, respectively. This suggests that the trained FE surrogate model possesses a high prediction accuracy. To find the optimal frame design factors that minimize both deflection and weight, we first generated 1 million datasets within the level range of frame design factors and then input them into the trained model to obtain predicted values. Using the same computing resources as those used for the FEA, the time taken to generate 1 million datasets with the trained FE surrogate model was 0.957 s, and the time taken to predict the deflection and weight values for 1 million datasets was 72.014 s. Subsequently, we scaled the predicted values for deflection and weight to between 0 and 1 using min–max normalization^15^. Fig. 1c shows the relationship between deflection and weight for 1 million datasets. As illustrated in the Fig. 1c, deflection and weight have an inverse relationship, where a decrease in one results in an increase in the other. Therefore, when the sum of the normalized values of the deflection and weight set is minimized, it can be considered that both deflection and weight are minimized. As a result, the factor values that minimize both deflection and weight were found to be a = 1.5176, b = 13.7105, c = 1.5012, d = 2.9898, e = 4.3123. It took 0.264 s to find the optimal values. At this point, the deflection was 11.1 mm and the weight was 3.6 kg. Table 1 shows the frame shape factors before and after minimization of deflection and weight. The optimal frame design factor values have been adjusted to the first decimal place, considering future manufacturing processes. The frame shapes before and after optimization are illustrated in the Fig. 1e.

**Table 1:** The values of the existing and optimal frame design factors.

	a	b	c	d	e
Existing (mm)	1.5	8.0	1.5	1.7	2.5
Optimal (mm)	1.5	13.7	1.5	3.0	4.3

After modifying the PV module frame with the optimal factors identified through the FE surrogate model, a FEA was performed. The results showed a deflection of 11.1 mm and a weight of 3.6 kg. Compared to the predictions of the FE surrogate model, both deflection and weight matched. The Table 2 presents the deflection and weight under the existing and optimal conditions. The weight increased by 0.4 kg, approximately 12.8% increase, while the deflection decreased by 1.2 mm, approximately 9.6% reduction when comparing the results of the existing and optimal conditions.

**Table 2:** Comparison of deflection and weight.

	$$\delta$$ (mm)	*w* (kg)
Existing	12.3	3.2
Optimal	11.1	3.6

As a result, we derived an optimal set of deflection and weight where both values are appropriately minimized within their relationship. However, this does not necessarily mean that these conditions will guarantee the PV module’s optimal efficiency. Additionally, even if the weight increases, reducing the deflection can decrease the impact on cell damage, and even with some deflection, reducing the weight can offer cost advantages. This is a consideration for designers between the efficiency and cost of PV module. Therefore, by analysing the relationship between each factor’s deflection and weight, we aim to offer guidance on the selection of PV module frame design factors.

**Figure 2:**
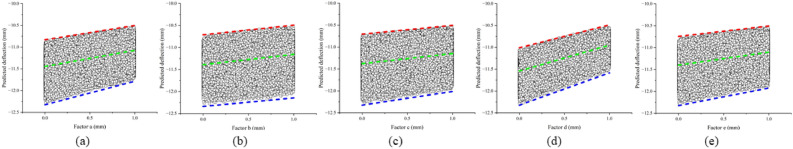
Distribution of FE surrogate model predictions for deflection by frame design factor (**a**) a (**b**) b (**c**) c (**d**) d (**e**) e.

**Figure 3:**
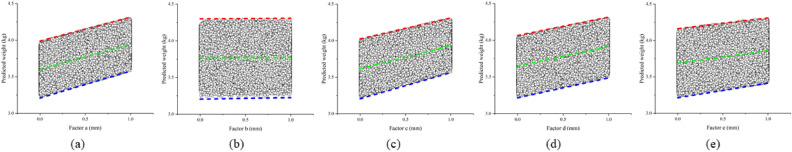
Distribution of FE surrogate model predictions for weight by frame design factor (**a**) a (**b**) b (**c**) c (**d**) d (**e**) e.

Figures 2 and 3 display the distribution of deflection and weight for one million datasets predicted by the FE surrogate model, categorized by each frame design factor. The red line at the upper boundary of the distribution represents the trend line for predictions obtained by generating and inputting 100,000 datasets within the range of the variable factor, with all other factors at their maximum values, into the DNN training model. Similarly, the blue line at the lower boundary represents the trend line for predictions with all other factors at their minimum values, excluding the variable factor. The green line in the middle represents the trend line for predictions when all other factors are at their median values. To compare the slopes of the trend lines within the factor range, each factor was standardized through min-max scaling. The slopes of the trend lines for each factor are presented in the Table 3. As can be seen in the Fig. 2 and Table 3, the factor with the greatest reduction in deflection as its dimension increases was identified as d, followed by factors a, e, c, b in order. Based on the slopes of the trend lines, it appears that an increase in dimension when other factors are at their minimum values results in a greater reduction in deflection than when other factors are at their maximum values. In the Fig. 3, it can be observed that the weight increases with the dimensions of all factors except for b. Factor b, being related to the position, did not influence the weight with its changes. The factor that had the most significant impact on weight due to dimensional changes was identified as a, followed by c, d, e, in order. This correlates with the increase in area due to changes in dimensions. The slope values of the trend lines for weight, when other factors were at their maximum, median, and minimum values, appeared relatively consistent compared to the slope values for deflection. In predicting the optimal values of frame factors through the DNN surrogate model, a and c were derived as minimum values, d as the maximum value, and b and e as values close to the maximum. Based on these results, it appears reasonable that a and c, which exhibit a larger increase in weight relative to other factors with dimension increases, are at their minimum values. It also seems justifiable that d, which shows the greatest increase in deflection but has a moderate increase in weight, is derived as the maximum value. Furthermore, factor e appears to have been slightly adjusted from the maximum value due to its similar trend to d but relatively smaller impact on the outcome. For factor b, which does not influence weight and only affects deflection, being derived as a maximum value seems preferable. However, interactions among other factors or errors in the learning process might result in a slightly reduced deflection at the maximum value, leading to its derivation as a slightly lower value than the maximum. Based on this information, it is believed that designers can derive the optimal frame design factors to achieve their prioritized outcomes, whether it be stability and performance or cost, among others. Our study is limited to frame design and does not address material types or the thickness of other components. So, if our approach using the developed FE surrogate model is applied to optimize other factors, it is expected that more efficient PV module development can be achieved.

**Table 3:** Slope of the trend line for deflection and weight for each factor.

	a		b		$\textrm{c}$		$\textrm{d}$		e	
	$\delta$	*w*	$\delta$	*w*	$\delta$	*w*	$\delta$	*w*	$\delta$	*w*
Maximum	0.54	0.37	0.19	0.02	0.31	0.36	0.74	0.27	0.40	0.20
Median	0.37	0.34	0.23	0.00	0.23	0.32	0.59	0.26	0.30	0.17
Minimum	0.33	0.33	0.22	0.01	0.20	0.29	0.52	0.25	0.24	0.15

## Conclusion

In this study, we detailed a method for optimizing the frame of large-scale bifacial PV module to improve deflection, utilizing a DNN-based FE surrogate model. We proposed a method for developing a FE model under mechanical load conditions for large-scale bifacial PV module. Based on those results, we successfully designed an FE surrogate model capable of predicting outcomes of FEA. The FE surrogate model was designed based on DNN, and the optimal hyperparameters were derived using the Bayesian optimization method, with accuracy improved through the k-fold cross-validation method. As a result, the FE surrogate model demonstrated high accuracy for a dataset of 243, with the MAPE and $R^2$ values for deflection and weight being an average of 0.0017, 0.9972 for the training set, and 0.0020, 0.9962 for the test set, respectively. We generated 1 million datasets within the factor range and used the trained model to obtain the predicted values. From these, we derived the optimal factors that minimize deflection and weight: a = 1.5, b = 13.7, c = 1.5, d = 3.0, e = 4.3. Under these conditions, the deflection was 11.1 mm, and the weight was 3.6 kg. When performing FEA with these optimal factors, the deflection and weight had almost no error. Compared to the existing frame design, the weight increased by approximately 12.8%, while the deflection decreased by about 9.6%. Generating the 1 million datasets took 0.957 s, obtaining the predicted deflection and weight values for these datasets took approximately 72.014 s, and finding the optimal values from these predictions took about 0.264 s. In contrast, using traditional FEA to obtain deflection and weight for a single case took about 4800 s. Additionally, the impact of each factor’s variations on deflection and weight was analyzed. Furthermore, by discussing the optimal results of the FE surrogate model, guidance for selecting the optimal frame factors was provided. Consequently, our FE surrogate model identified the optimal design factors that minimize deflection while minimally increasing weight compared to the existing frame. Additionally, by accurately predicting deflection and weight for these factors, it is expected to significantly reduce the time and cost required for PV module frame design. We conducted optimization only for the frame shape of large-scale bifacial PV module, without considering materials or thicknesses of other components. Therefore, it is expected that our study can be utilized for the efficient development of large-scale bifacial PV modules in the future by applying optimization for thicknesses and properties of each material to enhance module stiffness.

## Experimental procedure

### Structure of large-scale bifacial PV module

**Figure 4:**
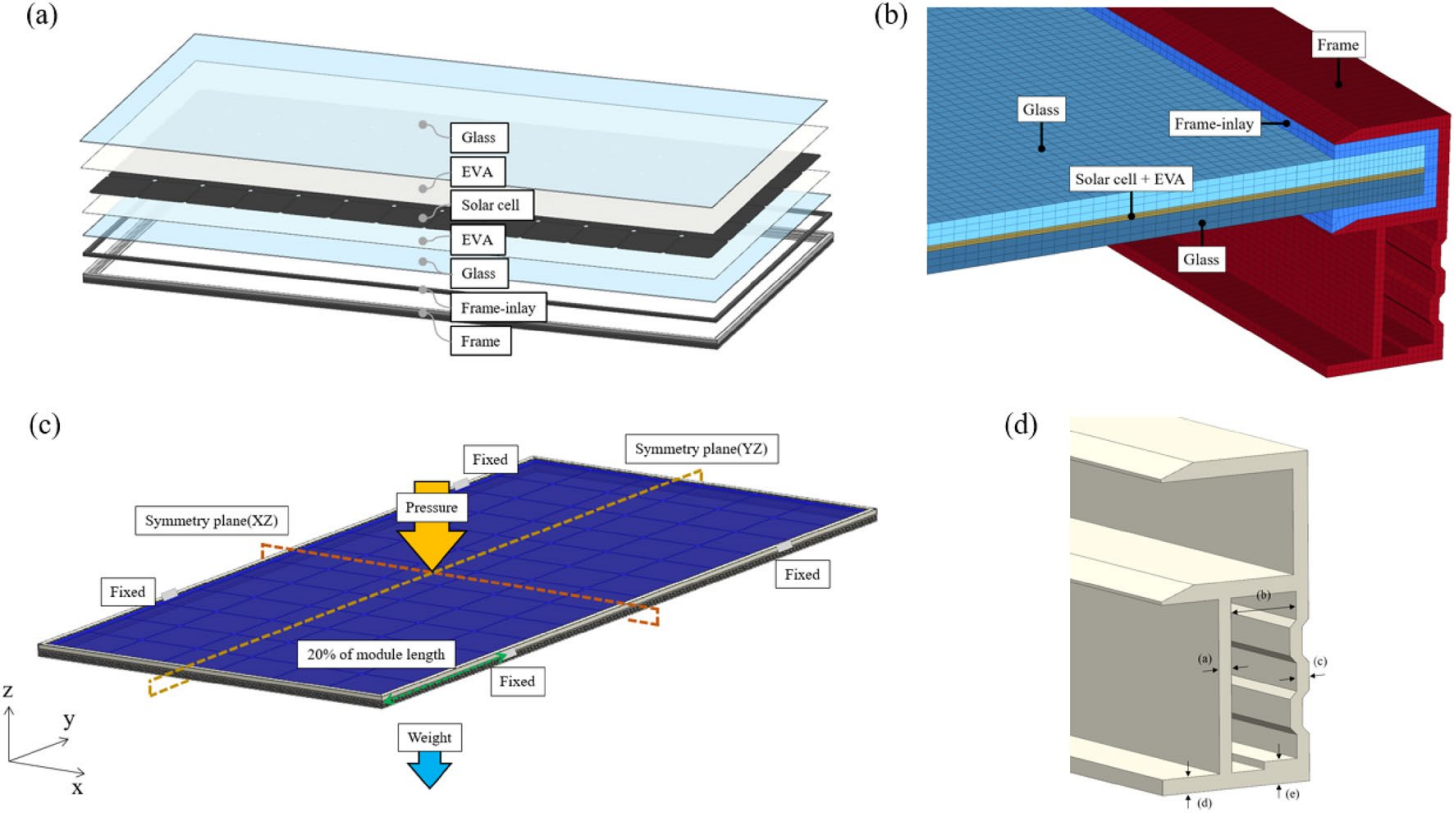
(**a**) Structure and components of large-scale bifacial PV module. (**b**) Cross-section of mesh modeling for a large-scale PV module. (**c**) Boundary conditions for FEA. (**d**) Design factors of PV module frame.

For the design optimization of the frame of large-scale bifacial PV module, we referred to a 585W-rated bifacial PV module containing a total of 78 M10 cells ($182\times 182$ mm$^2$) arranged in a $6\times 13$ configuration^16^. The module components consist of silicon cells, EVA, glass, frame, and frame-inlay, as illustrated in Fig. 4a. The thickness of each component is specified in Table 4. A cell thickness of 0.18 mm was chosen, and the post-lamination thickness was set to 0.8 mm^17^. The thickness of the glass placed on both sides is 3.2 mm, and the thickness of the frame-inlay is 2.0 mm. The spacing between cells is set at 2.0 mm, and the distance between cells and the frame is chosen as 21.0 mm^2,18^.

**Table 4:** Thickness of components in large-scale bifacial PV module..

Layer	Thickness (mm)
Glass	3.2
Solar cell	0.18
EVA + Solar cell	0.8
Frame-inlay	2.0

### Mechanical properties and FE model

The mechanical properties of each component in the large-scale bifacial PV module are provided in Table 5. The tensor representation of isotropic material according to Hooke’s law is expressed as in Eq. (1). The silicon wafer used in this study is a single-crystal solar wafer with the surface normal along the <100> plane. In this case, the stiffness matrix can be represented as in Eq. (2), and the applied coefficients in this study are $C_{11}$=164.8 GPa, $C_{12}$=63.5 GPa, $C_{44}$=79.0 GPa^12,13,19,20^.

**Table 5:** Mechanical properties of components in large-scale bifacial PV module.

Material	Density (kg/m$^3$)	Young’s modulus (GPa)	Poisson’s ratio
Glass	2500	70	0.2
EVA	0.96	T-dep.	0.4
Solar cell (Silicon)	2.329	Stiffness matrix	
Frame (Al)	2.7	70	0.33
Frame-inlay	0.067	0.0074	0.3

1 $$\begin{aligned}{}&\begin{Bmatrix} \varepsilon _{x} \\ \varepsilon _{y} \\ \varepsilon _{z} \\ \gamma _{xy} \\ \gamma _{yz} \\ \gamma _{zx} \\ \end{Bmatrix} = \begin{bmatrix} \frac{1}{E} &{} \quad -\frac{\nu }{E} &{} \quad -\frac{\nu }{E} &{} \quad 0 &{} \quad 0 &{} \quad 0 \\ -\frac{\nu }{E} &{} \quad \frac{1}{E} &{} \quad -\frac{\nu }{E} &{} \quad 0 &{} \quad 0 &{} \quad 0 \\ -\frac{\nu }{E} &{} \quad -\frac{\nu }{E} &{} \quad \frac{1}{E} &{} \quad 0 &{} \quad 0 &{} \quad 0 \\ 0 &{} \quad 0 &{} \quad 0 &{} \quad \frac{1}{G} &{} \quad 0 &{} \quad 0 \\ 0 &{} \quad 0 &{} \quad 0 &{} \quad 0 &{} \quad \frac{1}{G} &{} \quad 0 \\ 0 &{} \quad 0 &{} \quad 0 &{} \quad 0 &{} \quad 0 &{} \quad \frac{1}{G} \\ \end{bmatrix} \begin{Bmatrix} \sigma _{x} \\ \sigma _{y} \\ \sigma _{z} \\ \tau _{xy} \\ \tau _{yz} \\ \tau _{zx} \\ \end{Bmatrix} \end{aligned}$$

2 $$\begin{aligned}{}&\begin{pmatrix} C_{11} &{} \quad C_{12} &{} \quad C_{12} &{} \quad 0 &{} \quad 0 &{} \quad 0 \\ C_{12} &{} \quad C_{11} &{} \quad C_{12} &{} \quad 0 &{} \quad 0 &{} \quad 0 \\ C_{12} &{} \quad C_{12} &{} \quad C_{11} &{} \quad 0 &{} \quad 0 &{} \quad 0 \\ 0 &{} \quad 0 &{} \quad 0 &{} \quad C_{44} &{} \quad 0 &{} \quad 0 \\ 0 &{} \quad 0 &{} \quad 0 &{} \quad 0 &{} \quad C_{44} &{} \quad 0 \\ 0 &{} \quad 0 &{} \quad 0 &{} \quad 0 &{} \quad 0 &{} \quad C_{44} \\ \end{pmatrix} \end{aligned}$$

The cross-sectional mesh model for the PV module for FEA is shown in Fig. 4b. Eight-node hexahedral elements (C3D8) were utilized, and contact between elements was simulated by node coupling, assuming a frictionless and fully bonded connection. The total number of nodes in the FE model for case 1 is 2,302,520, and the number of elements is 2,038,205. FEA was performed using the commercial software ABAQUS/STANDARD R2017.

### Boundary conditions

FEA was conducted based on the mechanical load test method of crystalline silicon solar cells specified in the International Electrotechnical Commission (IEC) international standard (IEC 61215). According to the mechanical load test method, it is assumed that at a wind speed of 130 km/h, the wind pressure corresponds approximately to ±800 Pa. The test specifies the application of a safety factor of 3, resulting in a load of 2400 Pa applied to the front of the module^21^. Figure 4c illustrates the boundary conditions used for the FEA conducted in this experiment. To expedite the analysis time, a symmetry condition was applied, and FEA was performed for a 1/4 model. The distributed load on the front of the module, considering the self-weight due to gravity, was incrementally applied up to 600 Pa. The constraint location was applied at approximately 20 % of the module length from the module’s end, where bolts were fastened.^7^

### Design of experiment

To identify the factors playing a role in supporting the frame when subjected to loads in determining the shape of the Al frame for PV module, we conducted FEA using a commercially available Al frame. Based on the stress concentration and deformation locations identified in the FEA results, we determined the design factors for the frame as shown in Fig. 4d. We established a total of 243 experimental plans by arranging five frame design factors into three levels each. The level table for the factors used is presented in Table 6.

**Table 6:** Level of design factors.

Factor	Level (mm)		
a	1.5	2.0	2.5
b	8.0	11.0	14.0
c	1.5	2.0	2.5
d	1.7	2.3	3.0
e	2.5	3.7	5.0

### Finite element surrogate model

**Figure 5:**
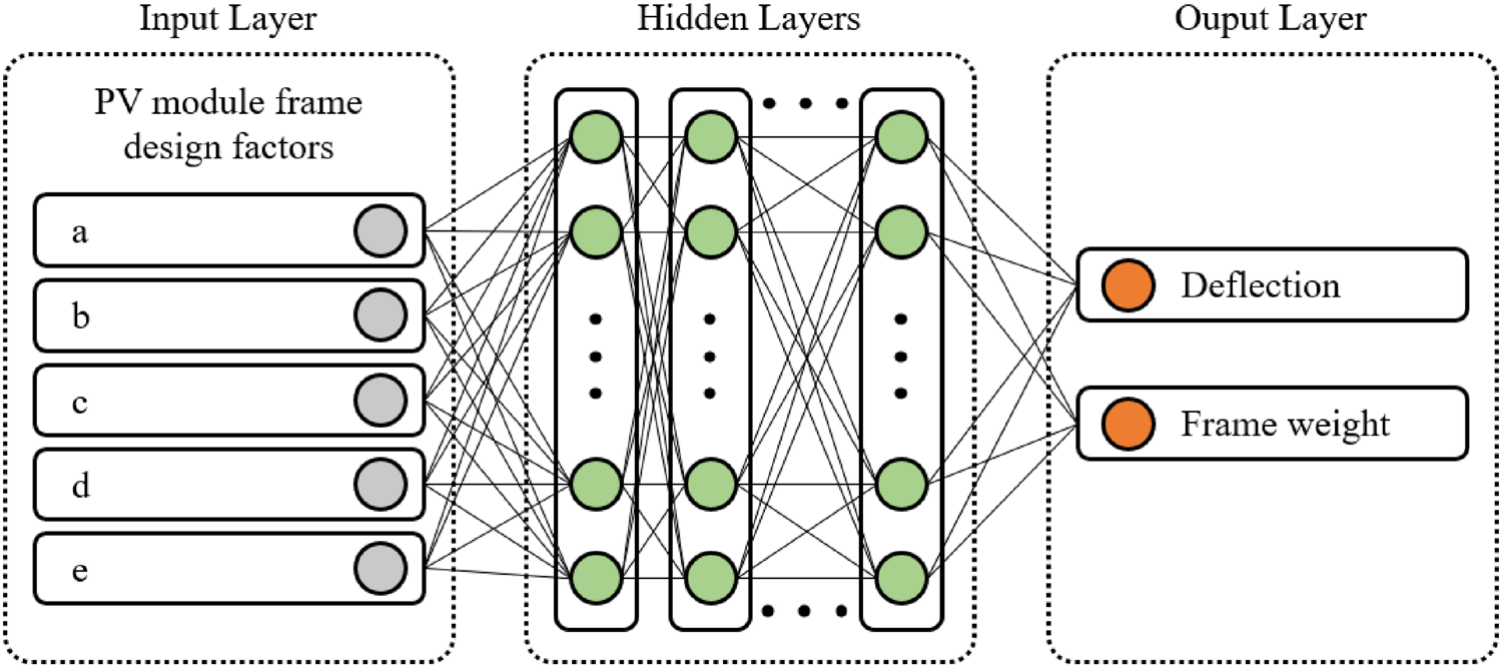
Schematic diagram of the DNN for PV module surrogate model.

FEA can demand significant computational resources, especially for large-scale high-fidelity FE models, due to the need for repeated executions to update the model^22^. Surrogate models are data-driven and can be used as fast emulators for FE models to facilitate a quicker model update process^23^. The core idea is to replace the high evaluation cost of the FE model, either wholly or partially, with a surrogate model that has a lower evaluation cost for the purpose of updates. Surrogate modeling can be used for model calibration, identifiability and sensitivity analysis, uncertainty quantification, reliability analysis, design optimization, and any purpose that requires iterative model evaluations^24–27^. Thus, we aimed to develop a FE surrogate model based on deep learning methods, and the schematic diagram is as shown in Fig. 5. DNN is an advanced version of artificial neural network with multiple hidden layers between the input and output layers^28^. For the FE surrogate modeling, 243 FEA data points were applied as input data to the DNN algorithm. The input data were randomly split into two parts: a training set and a test set, with a ratio of 9:1^29,30^. To achieve more accurate predictions with the DNN, the k-fold cross-validation method was used to increase the amount of learning within the limited data set, with the applied k-value being 10^31,32^.

### Hyperparameter tunning

DNN-based surrogate modeling involves finding an approximate function that minimizes the loss function for the given data. This can be optimized by adjusting hyperparameters such as learning rate, epochs, number of layers, number of nodes, and batch size, to ensure the loss function is minimized^33–35^. Bayesian optimization is an effective method for solving functions that are computationally expensive to find extrema^36^. The goal, as shown in Eq. (3), is to find the optimal solution where the unknown objective function *f* is maximized. Bayesian optimization is derived from Bayes’ theorem^36,37^, and as indicated in Eq. (4), given evidence data E, the posterior probability P(M|E) of a model M is proportional to the likelihood P(E|M) of observing E given model M, multiplied by the prior probability P(M). Where, A represents the search space of x. Therefore, Bayesian optimization is a probabilistic method for posterior estimation that utilizes prior distributions and sample information to predict subsequent values. It is known to employ a Kriging model, also known as Gaussian process model, for fitting the data and updating the posterior distribution.^38^

3 $$\begin{aligned}{}&x^+ = arg\, \max _{x \in a} f(x) \end{aligned}$$

4 $$\begin{aligned}{}&P(M|E)\; \propto \;P(E|M) \, P(M) \end{aligned}$$

Bayesian optimization obtains the posterior distribution through a Kriging model and uses the acquisition function to find the optimal solution where the objective function is maximized. The process of finding the optimal solution includes strategies for exploiting areas near observed high values, anticipating that a maximum exists there and exploring areas of highest uncertainty, anticipating that a maximum might be found there. The former can be realized by minimizing the Kriging prediction function, while the latter can be achieved by maximizing the Kriging uncertainty function^39^. Expected Improvement (EI) criterion, serving as the acquisition function, effectively balances the two strategies of exploitation and exploration^40^. Due to these advantages, EI is frequently used within the frameworks of Bayesian optimization^41^. Therefore, we applied the acquisition function EI to perform Bayesian optimization, deriving hyperparameters that minimize the loss function. Table 7 shows the hyperparameters to be optimized and their range of values.

**Table 7:** Range of values for hyperparameters..

Hyperparameter	Range of values
Number of hidden layers	2–5
Number of nodes in each hidden layer	1–128
Batch size	1–243
Learning rate	0.001–0.1

### Supplementary Information

Supplementary Information 1.

Supplementary Information 2.

## Data Availability

The datasets are available from the corresponding author upon reasonable request.
